# The breakthrough paradox: How focusing on one form of innovation jeopardizes the advancement of science

**DOI:** 10.15252/embr.202254772

**Published:** 2022-05-27

**Authors:** Ruth Falkenberg, Maximilian Fochler, Lisa Sigl, Hermann Bürstmayr, Stephanie Eichorst, Sebastian Michel, Eva Oburger, Christiana Staudinger, Barbara Steiner, Dagmar Woebken

**Affiliations:** ^1^ Research Platform Responsible Research and Innovation in Academic Practice University of Vienna Vienna Austria; ^2^ Department of Science and Technology Studies University of Vienna Vienna Austria; ^3^ Department of Agrobiotechnology University of Natural Resources and Life Sciences Tulln Austria; ^4^ Department of Microbiology and Ecosystem Science University of Vienna Vienna Austria; ^5^ Institute of Soil Research University of Natural Resources and Life Sciences Vienna Austria

**Keywords:** Careers, Economics, Law & Politics, Science Policy & Publishing

## Abstract

Research needs a balance of risk‐taking in “breakthrough projects” and gradual progress. For building a sustainable knowledge base, it is indispensable to provide support for both.

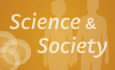

Science is about venturing into the unknown to find unexpected insights and establish new knowledge. Increasingly, academic institutions and funding agencies such as the European Research Council (ERC) explicitly encourage and support scientists to foster risky and hopefully ground‐breaking research. Such incentives are important and have been greatly appreciated by the scientific community. However, the success of the ERC has had its downsides, as other actors in the funding ecosystem have adopted the ERC’s focus on “breakthrough science” and respective notions of scientific excellence. We argue that these tendencies are concerning since disruptive breakthrough innovation is not the only form of innovation in research. While continuous, gradual innovation is often taken for granted, it could become endangered in a research and funding ecosystem that places ever higher value on breakthrough science. This is problematic since, paradoxically, breakthrough potential in science builds on gradual innovation. If the value of gradual innovation is not better recognized, the potential for breakthrough innovation may well be stifled.

While continuous, gradual innovation is often taken for granted, it could become endangered in a research and funding ecosystem that places ever higher value on breakthrough science.

Concerns that the hypercompetitive dynamics of the current scientific system may impede rather than spur innovative research have been voiced for many years (Alberts *et al*, 2014). As performance indicators continue to play a central role for promotions and grants, researchers are under pressure to publish extensively, quickly, and preferably in high‐ranking journals (Burrows, 2012). These dynamics increase the risk of mental health issues among scientists (Jaremka *et al*, 2020), dis‐incentivise relevant and important work (Benedictus *et al*, 2016), decrease the quality of scientific papers (Sarewitz, 2016) and induce conservative and short‐term thinking rather than risk‐taking and original thinking required for scientific innovation (Alberts *et al*, 2014; Fochler *et al*, 2016). Against this background, strong incentives for fostering innovative and daring research are indispensable.

## Breakthrough research is only one ingredient of successful science

Funding schemes such as the ERC, which explicitly support researchers to pursue more risky projects over a longer time span are therefore crucial for producing scientific breakthroughs. Yet, we argue that such funding schemes, as important as they are, are not sufficient on their own to foster scientific innovation. While it is essential to free researchers to prioritize long‐term thinking and to incentivize unconventional, daring research, scientific innovation does not necessarily come about in a “disruptive” or “ground‐breaking” manner in the frame of a single research project.

… scientific innovation may at times require continuous and often tedious work for a longer time period, rather than only innovative first leaps into novel areas.

This article is based on a collaboration between social and natural scientists in the framework of a science and technology studies research project. The central question was to identify conditions that foster or inhibit excellent basic research that addresses environmental problems. The social scientists conducted more than 70 interviews with researchers from the crop and soil‐related sciences and organized focus groups around specific issues. Central topics in these interviews were the dynamics and forms of innovation in science. An important outcome was the essential role that a variety of forms of innovation play for scientific progress, including work that may not, at first sight, be described as “ground‐breaking.”

As Falkenberg (2021) described elsewhere in more detail, scientific innovation may at times require continuous and often tedious work for a longer time period, rather than only innovative first leaps into novel areas. This applies to individual research groups but is even more true at the level of whole research fields and the collective knowledge they represent. Especially inter‐ and transdisciplinary collaborations that are now widely considered as leading to highly innovative findings need time to form until they can produce ground‐breaking results (Brown *et al*, 2015). As such, research that “only” follows up on previous findings, and that may at first sight seem incremental, is indispensable. It is not new to say that current science stands on the shoulders of giants—yet the implications sometimes seem to be forgotten.

## “Normal science” may be pushed into a tight corner

Of course, it is not about either breakthroughs or continuity. In an ideal system, both should be in productive tension and interaction along with funding and institutional conditions that create space both for risky research with “breakthrough potential,” and gradual and continuous science that lays the ground for important innovations. The historian and philosopher of science Thomas Kuhn (1962) was among the first to distinguish between “normal science,” building knowledge along a paradigm, and “scientific revolutions,” which shift the paradigm and allow for new venues of thinking and research. However, paradigm shifts in the Kuhnian sense are rare and not likely to be the result of single projects. When talking to researchers about what is required for meaningful progress and innovations in their field, they rather describe a balance between what Kuhn would call “normal science” and high‐risk leaps into the unknown. The latter may turn out to produce breakthrough innovations or may as well fail in producing meaningful results at all.

“Normal science” […] has been increasingly disregarded with a growing focus on “outstanding,” “excellent,” and “frontier” science, first in the United States and later in European science policy.

Many of the researchers with whom we engaged in our project felt that the “normal” part in this scheme is increasingly under pressure. This reflects the argument by historians of science and science policy scholars that Kuhn and his colleagues in the philosophy, history, and social studies of science have not had much influence on how science funding was institutionalized during the 20^th^ century (Flink & Peter, 2018). “Normal science” in particular has been increasingly disregarded with a growing focus on “outstanding,” “excellent,” and “frontier” science, first in the United States and later in European science policy. During the past decades, third‐party funding, initially seen as complementing “normal science,” has strongly gained importance at the expense of institutional basic funding. This now undermines the capacity of many research groups to perform “normal science.” As Flink and Peter (2018) show, the founding and success of the ERC build on the discursive legacy of stressing the importance of disruptive and transformative innovation in science.

## Is breakthrough research becoming the new norm?

There is little doubt that the work of the ERC has had an enormous positive impact on science in Europe and beyond (König, 2017). However, the ERC and its funding model were conceptualized as complementing existing funding ecosystems and not as setting a new norm for good research. We argue that there is a danger that this role balance in the funding ecosystem may be shifting, paradoxically because of the success of the ERC. During the past decade, ERC funding and ERC definitions of quality have increasingly become seen as the gold standard of excellence in European academia (König, 2017). Faced with growing numbers of grant applications and limited budgets, national funders increasingly prioritize research that promises to daringly venture into novel areas. In Austria, for example, the Austrian Science Fund (FWF), the central funder of basic research, explicitly notes in their guidelines for standalone projects “that the next logical step or the incremental further development of published data is not considered to be innovative or original” (p. 8)—hence not worth being funded. Other European countries show similar tendencies. The Independent Research Fund Denmark, for example, writes in their call for proposals that applications should “display innovative research as opposed to expanding on already ongoing research” (p. 19). What is particularly problematic about these ERC‐like demands for innovative breakthrough research is that they are usually matched neither with financial support comparable to an ERC grant, nor with longer‐term prospects, thus making the development of breakthrough innovations in a single research project very unlikely.

… the ERC and its funding model were conceptualized as complementing existing funding ecosystems and not as setting a new norm for good research.

In addition, ERC funding and funding from grant agencies with a comparable focus on excellence and breakthrough research have become a major factor in hiring and tenure decisions (Scholten *et al*, 2021). In planning their projects, researchers anticipate requirements for ERC‐like funding that is oriented towards breakthrough science, thus potentially de‐prioritizing other lines of research that may be equally valuable. As Scholten *et al* (2021) stress, this type of excellence funding has strong positive effects on the few researchers and groups that succeed, in the sense that they can build “protected spaces” within the university (Gläser *et al,* 2014). However, these positive effects are counteracted by the negative effects for the many other researchers who do not receive such funding but still neglect more gradual lines of research.

## Too many barriers to longer‐term, continuous research

If the majority of funders turned to support breakthrough and transformative research as a priority, and if promotion committees only valued applicants who have made frequent moves of self‐reinvention and turned to novel topics within their career, this may impede other kinds of valuable research that are equally required for scientific innovation and progress. The dominant precarious employment situation for junior researchers already creates an environment where they constantly need to change projects and topics which hinders continuous work (Dirnagl, 2022). Ultimately, the progress of science is impaired by a lack of funding for research to build an in‐depth knowledge base. Especially inter‐ and transdisciplinary projects that are considered to be urgently needed when it comes to addressing social‐environmental problems but need continuous engagement and building of collaboration may also be disrupted or dis‐incentivised (Brown *et al*, 2015).

Even in purely economic terms, it is inefficient and wasteful if promising lines of research cannot be pursued further and brought to a satisfactory end…

Even in purely economic terms, it is inefficient and wasteful if promising lines of research cannot be pursued further and brought to a satisfactory end because research groups lack stable funding for doing so. We call this the breakthrough paradox: The currently strong emphasis on scientific breakthroughs sometimes seems to hinder the often tedious and long‐lasting research that is indispensable for such breakthroughs.

## Implications for research governance

What does this imply for research governance in terms of concrete recommendations for funding science? Scientific innovation comes in multiple forms and therefore requires a plurality of funding schemes, and ultimately a plurality of criteria by which researchers are evaluated (Hicks *et al*, 2015). Creating possibilities for blue‐sky, breakthrough‐oriented research through ERC‐like funding is essential. However, institutions that fund, value, and support continuity are just as important in providing and sustaining “protected spaces” for risk‐taking and innovation.

What is required to guarantee such a balance in the funding ecosystem? First, the sketched dynamics are strongly exacerbated by the increasing volatility of and competition for funding in many academic systems. More stable and generous funding on all levels must be the basis for any functioning research funding ecosystem.

Second, it requires coordination in the funding ecosystem rather than institutional isomorphism and copying the currently most prestigious model. While institutions such as the ERC are highly valuable, it is indispensable that others, such as national funders, maintain a different focus and support continuous science. All funding institutions need to explicitly reflect on their role in the wider funding ecosystem and to be mindful of the importance of continuous innovation processes in science. In addition, it is imperative that funders make their perception and their corresponding definitions of key terms such as “excellence” and “quality” explicit in guidelines and communication with reviewers. There is a danger that reviewers may operate on different implicit understandings of these terms and in question resort to dominant role models in the absence of guiding principles.

Scientific innovation comes in multiple forms and therefore requires a plurality of funding schemes and ultimately a plurality of criteria by which researchers are evaluated.

Third, not only funding institutions but also research institutions and universities should consider the importance of continuity in building knowledge bases. The right balance of disruptive innovation and continuity is a topic of wider relevance for the dynamics of knowledge production in contemporary societies and not only an issue of research funding. It is indispensable to do greater justice to this balance, in order to escape the breakthrough paradox that we are currently facing and to foster sustainable innovation.

## Disclosure and competing interests statement

The authors declare that they have no conflict of interest.
